# Coenzyme Q10 and Degenerative Disorders Affecting Longevity: An Overview

**DOI:** 10.3390/antiox8020044

**Published:** 2019-02-16

**Authors:** David Mantle, Iain Hargreaves

**Affiliations:** 1 Pharma Nord (UK) Ltd., Telford Court, Morpeth, NE61 2DB Northumberland, UK; 2 School of Pharmacy, Liverpool John Moores University, L3 5UA Liverpool, UK; i.hargreaves@ucl.ac.uk

**Keywords:** coenzyme Q10, oxidative stress, inflammation, diabetes, cardiovascular disease, chronic kidney disease and liver disease, mitochondria

## Abstract

Longevity is determined by a number of factors, including genetic, environmental and lifestyle factors. A major factor affecting longevity is the development of degenerative disorders such as cardiovascular disease, diabetes, kidney disease and liver disease, particularly where these occur as co-morbidities. In this article, we review the potential role of supplementation with coenzyme Q10 (CoQ10) for the prevention or management of these disorders. Thus, randomised controlled clinical trials have shown supplementation with CoQ10 or CoQ10 plus selenium reduces mortality by approximately 50% in patients with cardiovascular disease, or in the normal elderly population, respectively. Similarly, CoQ10 supplementation improves glycaemic control and vascular dysfunction in type II diabetes, improves renal function in patients with chronic kidney disease, and reduces liver inflammation in patients with non-alcoholic fatty liver disease. The beneficial role of supplemental CoQ10 in the above disorders is considered to result from a combination of its roles in cellular energy generation, as an antioxidant and as an anti-inflammatory agent.

## 1. Introduction

**Figure 1:**
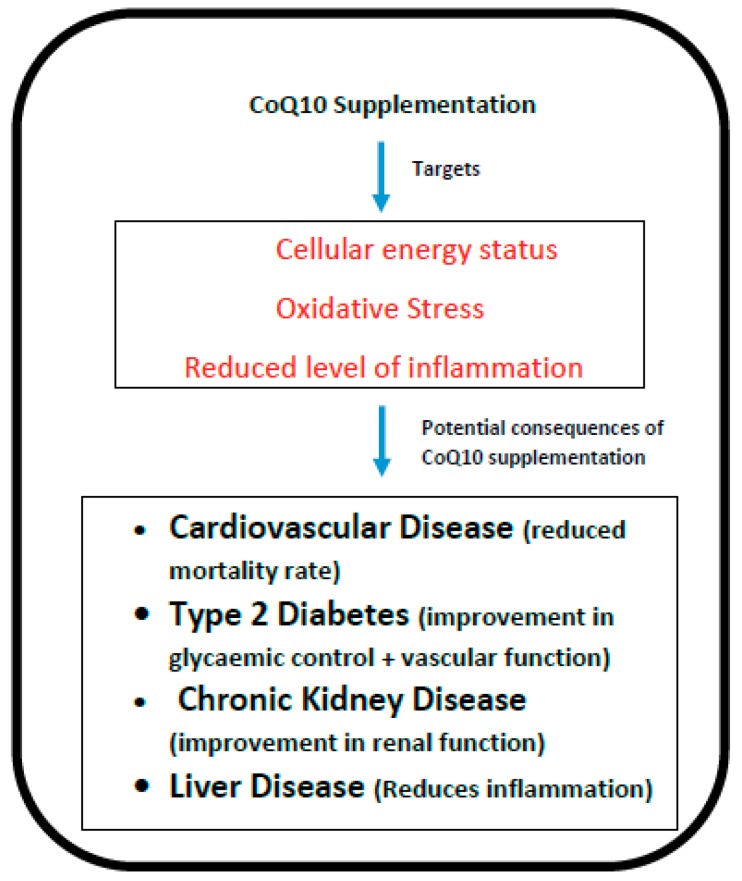
The potential targets and consequences of coenzyme Q10 (CoQ10) supplementation.

Longevity depends on a number of factors, one of the most important determinants being the development of chronic degenerative diseases, particularly where these occur as co-morbid conditions. For example, a 70-year old individual with no chronic degenerative diseases will be expected to live for more than 20 years, whereas those with several such disorders would be predicted to live some 8 years fewer [1]. In the present article, we have reviewed how oral supplementation with coenzyme Q10 (CoQ10) may significantly benefit degenerative disorders such as cardiovascular disease, diabetes, kidney disease and liver disease, thereby promoting longevity. The rationale for CoQ10 supplementation in these disorders is based on its key role in cellular metabolism; in addition to its role in cellular energy generation, CoQ10 has antioxidant and anti-inflammatory action (Figure 1), is involved in DNA replication and repair (through its essential cofactor role in pyrimidine synthesis), regulates the physiochemical properties of cellular membranes, and modulates gene expression [2]. Meta-analyses of clinical studies have demonstrated that supplementation with CoQ10 significantly reduces levels of the inflammatory mediators C-reactive protein (CRP), interleukin-6 (IL-6) and tumour necrosis factor alpha (TNF-α) respectively [3,4]. Schmelzer et al. [5] had previously provided evidence that levels of inflammatory mediators such as CRP, IL-6 and TNF-α are reduced via the effect of supplementary CoQ10 on the nuclear transcription factor NF kappa beta. Most of the body’s daily CoQ10 requirement is derived from endogenous synthesis [6], and this is known to decline substantially with age [7]. This age-related decline in endogenous CoQ10 synthesis may therefore be directly linked to longevity via the development of these degenerative disorders [8].

## 2. Cardiovascular Disease

The role of CoQ10 supplementation in the treatment or prevention of cardiovascular disease has been detailed in previous reviews [9,10]. Although there is a distinct lack of high quality studies assessing the utility of CoQ10 supplementation in the primary prevention of cardiovascular disease [11], two recently published randomised, double blind, placebo controlled clinical trials have demonstrated the efficacy of supplementation with CoQ10 (Q-SYMBIO) or CoQ10 plus selenium (KISEL-10) in substantially reducing mortality risk in patients with heart failure, or in the normal elderly population, respectively.

The Q-SYMBIO study was carried out in some 400 patients with chronic heart failure (New York Heart Association; NYHA class III or IV), and the effect of CoQ10 supplementation (3 × 100 mg/day for two years) on symptoms and biomarker status (hence the trial acronym Q-SYMBIO) were assessed [12]. Assessment included clinical examination, echocardiography and pro-BNP(B-type natriuretic peptide) status (a biomarker related to cardiac wall tension and ejection fraction, used to quantitate heart failure). The primary long-term endpoint was time to first major adverse cardiovascular event (MACE), which included unplanned hospitalisation due to worsening heart failure and cardiovascular death. 

Supplementation with CoQ10 reduced the risk of MACE by 42%, with a reduction of approximately 40% in both cardiac related deaths and all-cause mortality. There was no significant difference in adverse events between the CoQ10 treated and placebo groups over the duration of the study. CoQ10 has therefore been reported as the first novel drug (i.e., one addressing cardiac myocyte energy depletion) to improve heart failure mortality in over a decade.

The KISEL-10 study was a double blind, randomised, placebo controlled clinical trial carried out on normal elderly individuals (70–88yrs) from the Kinda region of Stockholm [13]; 440 participants were supplemented with 200 mg/day coenzyme Q10 (CoQ10; Bio-Quinone 100mg) and 200 mcg/day selenium (SelenoPrecise), or placebo, over a five year period (hence the trial acronym KISEL-10). Clinical examination, echocardiography and biomarker measurements were carried out at six-month intervals. Supplementation with CoQ10 and selenium resulted in significant reductions in the blood levels of pro-BNP [14], C-reactive protein and, soluble platelet selectin (sP selectin) as markers of inflammation [15], and copeptin and adrenomedullin as markers of oxidative stress [16]. Quality of life was quantified using the Short Form-36 (SF-36), Cardiac Health Profile (CHP) and Overall Quality of Life (overall-QOL) questionnaires. Supplementation resulted in a significant reduction in the number of days in hospital and significantly slowed the deterioration in health related quality of life [17]. Echocardiography showed significantly better cardiac function in supplemented participants, whose risk of cardiovascular mortality was significantly reduced by 53% [14]. It is of note that a follow-up study showed the protective effect of CoQ10 and selenium supplementation in reducing the risk of cardiovascular mortality persisted for several years after the end of the intervention period [18].

The KISEL-10 study demonstrated long-term supplementation with CoQ10 and selenium significantly improved quality of life and heart function, and reduced hospitalisation frequency and the risk of cardiovascular related mortality in the elderly. At a mechanistic level, the benefits of supplementation with CoQ10 and selenium are derived both from their role in cellular energy production, and as tissue protecting antioxidants. Supplementation with CoQ10 and selenium may be of particular benefit for individuals prescribed statins, since the latter are known to interfere in the production of both CoQ10 and selenoproteins such as thioredoxin reductase and glutathione peroxidase [19]. Thioredoxin reductase is responsible for the interconversion of ubiquinone to ubiquinol, and glutatione peroxidase is an important antioxidant enzyme. 

The KISEL-10 study supplemented two nutrients, CoQ10 and selenium, with a key role in heart function, which were also likely to be deficient in the elderly population investigated. In the case of CoQ10, most of the body’s daily requirement is obtained by endogenous synthesis. As people age, the capacity for endogenous synthesis declines, such that blood CoQ10 levels in a 65 year old are approximately half that in a 25 year old. With regard to selenium, dietary intake in many European countries, including Sweden and the UK, is known to be sub-optimal, particularly in the elderly. The above provides a rationale for the success of the KISEL-10 protocol in reducing the risk of death from heart disease.

Amongst the normal elderly Swedish population, those with the lowest levels of blood selenium were found to be at increased risk of cardiovascular mortality [20]. The age-related decline in endogenous CoQ10 synthesis, sub-optimal dietary intake of selenium and associated increased risk of heart disease and cardiovascular mortality therefore provided the rationale for the supplementation regime employed in the KISEL-10 study.

## 3. Diabetes

The potential benefit of CoQ10 supplementation in type II diabetes has been recently reviewed [21], from which the following information has been summarized. Firstly, a number of studies have identified depleted blood CoQ10 levels in patients with type II diabetes, an example being the study by El-Ghoroury et al. [22].

Some 15 randomised controlled clinical trials supplementing CoQ10 (typically 100–200 mg/day for 3–6 months) in type II diabetic patients are currently listed by Medline. Although Eriksson et al. [23] found no significant benefit of CoQ10 supplementation on glycaemic control in type II diabetics, subsequent studies reported CoQ10 supplementation significantly improved fasting plasma glucose and HbA1c (glycated haemoglobin )levels [24,25,26,27,28].

Similarly, glycaemic control and blood antioxidant levels were significantly improved in type II diabetics following supplementation (100 mg/day for three months) with the reduced (ubiquinol) form of CoQ10 [29]. In patients with diabetic neuropathy, supplementation with CoQ10 (200 mg/day for three months) did not significantly benefit neuropathic symptoms, but reduced inflammation and increased insulin sensitivity [30]. Supplementation with CoQ10 in metabolic syndrome patients (100 mg/day for two months, [31]) or obese patients (200 mg/day for three months, [32]) improved glycaemic control, and in the latter case waist circumference. Yoo and Yum [33] suggested CoQ10 supplementation in patients with impaired glucose tolerance could slow the progression from pre-diabetes to overt type II diabetes. The benefit of CoQ10 supplementation on glycaemic control and blood lipid levels has been confirmed in a recent meta-analysis by Zhang et al. [34].

Supplemental CoQ10 may benefit type II diabetes via several mechanisms, for example by promoting enhanced levels of cellular energy required for glucose metabolism, or via direct modulation of the expression of genes relevant to glucose metabolism, or via its antioxidant action [35]. Thus, supplementation with CoQ10 (100 mg/day for three months) in patients with diabetic nephropathy significantly reduced inflammation via improved gene expression of peroxisome proliferators activated receptor gamma, interleukin-1 and tumour necrosis factor alpha [36]. With regard to antioxidant activity, evidence of increased oxidative stress (correlating with depleted blood CoQ10 levels) was reported by Ates et al. [37]. Randomised controlled trials supplementing CoQ10 (200 mg/day for three months) in type II diabetic patients found reduced blood levels of oxidative stress markers, improved endothelial function/blood flow and reduced cardiovascular risk [38,39]

The use of statins (particularly simvastatin) has been associated with an increased risk of between 10% and 40% of developing type II diabetes [40,41]; this is thought to result from statin-induced depletions of circulatory levels of CoQ10, adiponectin and glucose transporter-4 (GLUT4) protein [42]. Although CoQ10 administration has been shown to prevent simvastatin induced GLUT4 protein levels in cell culture [43], Kuhlman et al. [44] failed to find significant changes in muscle GLUT4 levels following supplementation with CoQ10 (400 mg/day for two months) in simvastatin treated subjects.

## 4. Chronic Kidney Disease (CKD)

The role of supplemental CoQ10 in chronic kidney disease (CKD) has been reviewed [45], from which the following information has been summarised. Plasma CoQ10 levels have been reported to be significantly lower in CKD patients (with or without haemodialysis), compared to normal controls [46,47,48]. There is evidence that CoQ10 supplementation may improve renal function and reduce the need for dialysis in patients with CKD. In a randomised controlled study [49], 97 CKD patients were given supplementary CoQ10 (3 × 100 mg daily for three months) or placebo. There was a significant improvement in markers of renal function (e.g., serum creatinine) in CoQ10 supplemented patients compared to placebo, in both dialysed and non-dialysed patients. In particular, the number of patients requiring dialysis in the CoQ10 treated group decreased from 21 to 12, whilst remaining unchanged at 24 in the placebo group. Decreased CoQ10 levels may be a particular issue in CKD patients prescribed statins, since some studies have reported a deficit in CoQ10 status in association with this pharmacotherapy in a subset of patients. It has been suggested that these patients may have some form of underlying mitochondrial disease and therefore may be more susceptible to the adverse effects of statin therapy [50]. 

Although haemodialysis is essential for removing uremic toxins, it is a consequence of the procedure that individuals are subject to additional oxidative stress (a result of neutrophil exposure to the synthetic material comprising the dialyser membrane), in addition to the oxidative stress associated with CKD. A number of clinical studies have reported that supplementation with CoQ10 significantly improves outcome in haemodialysis patients by reducing markers of oxidative stress and inflammation. In a randomised controlled trial, Zahed et al. [51] reported that CoQ10 supplementation (100 mg/day for three months) in end stage CKD patients undergoing haemodialysis significantly reduced serum levels of the inflammatory marker C-reactive protein. An open label dose escalation study by Yeung et al. [48] showed supplementation with CoQ10 over the range 300–1800 mg/day for 14 days to be safe and well tolerated, significantly reducing plasma levels of the oxidative stress marker isofuran.

Patients with CKD are at high risk of developing cardiovascular disease, with a 10–20 fold increased risk of cardiovascular mortality compared to non-CKD individuals. Overall, approximately 50% of deaths in CKD patients result from cardiovascular disease, rather than as a direct consequence of kidney failure. Conversely, cardiovascular disease can cause CKD leading to a vicious circle in which each disorder exacerbates the other. Thus, treatment of CKD can reduce the incidence of cardiovascular disease, and treatment of cardiovascular disease can reduce further deterioration in renal function. The ratio of plasma CoQ10 versus low density lipoprotein (LDL) cholesterol + VLDL (very low density lipoprotein) cholesterol, considered to be more important in athersclerosis prevention than the ratio of HDL (high density lipoprotein ):LDL cholesterol [52], was significantly lower in CKD patients (with or without dialysis) compared to controls [53]. A recent meta-analysis by Bakhshayeshkaram et al. [54] confirmed significant improvement in blood cholesterol, markers of oxidative stress, and creatinine levels following CoQ10 supplementation in CKD patients.

Epicardial fat thickness, a new risk factor for cardiovascular disease, was found to be significantly greater in CKD patients undergoing haemodialysis compared to controls and correlated with reduced plasma CoQ10 levels [55]. Similarly, coronary flow reserve, an indicator of atherosclerosis, was reported to be significantly lower in haemodialysis patients, correlating inversely with serum CoQ10 levels [55].

## 5. Liver Disease

Although endogenous CoQ10 synthesis occurs throughout the body, because of its physical size and high metabolic capacity, the liver is the major site of CoQ10 synthesis. In patients with liver disease where metabolic capacity has been compromised, a reduction in CoQ10 production is likely to have a deleterious effect on heart function. Thus, non-alcoholic fatty liver disease (NAFLD) is a risk factor for cardiovascular disease, which has been reported to be one of the major causes of death in NAFLD patients [56]. NAFLD is associated with heart failure, arrhythmias, valve dysfunction and atherosclerosis [57]. Alcohol-related liver disease is similarly associated with an increased risk of cardiovascular disorders; these include alcoholic cardiomyopathy, arterial hypertension and atrial fibrilation [58]. Reduced CoQ10 levels may be a particular problem in patients with fatty liver disease prescribed statins, since in addition to inhibiting cholesterol synthesis, statins also inhibit the production of CoQ10.

In addition to reducing the risk of cardiovascular problems in patients with liver disease, CoQ10 supplementation may also benefit the disease process within the liver by reducing inflammation and oxidative stress. For example, one of the principal mechanisms by which alcohol causes liver damage is via the generation of free radicals, and the antioxidant action of CoQ10 can help to protect liver cells from such oxidative damage [59]. Free radical induced oxidative stress has similarly been implicated in the pathogenesis of NAFLD [60].

A number of studies in animal models have demonstrated the ability of CoQ10 to reduce or prevent the development of liver cirrhosis following a variety of toxic insults; these include exposure to medicinal drugs [61], toxic chemicals [62] and parasitic microorganisms [63]. For example, in the study by Fouad and Jresat [60], the ability of CoQ10 to protect liver tissue against free radical induced oxidative damage was demonstrated; when acute liver injury was induced in rats via administration of acetaminophen (paracetamol), subsequent administration of CoQ10 reduced cirrhotic tissue damage via its antioxidant and anti-inflammatory action. Similarly, in rats prone to developing NAFLD, dietary supplementation with CoQ10 prevented further progression to cirrhosis via downregulation of markers of free radical induced oxidative stress and inflammation [64].

There have been relatively few clinical studies relating to CoQ10 and liver disease. Evidence for depleted CoQ10 blood levels in NAFLD patients was reported by Yessilova et al. [65]. Two randomized controlled clinical trials have been carried out to date supplementing CoQ10 in NAFLD patients; in both cases, 100 mg/day CoQ10 supplemented for four weeks [66] or 12 weeks [67] respectively resulted in significant reductions in blood markers for inflammation and liver damage.

## 6. CoQ10 Supplementation: Importance of Product Quality and Bioavailability

With regard to clinical studies involving oral supplementation with CoQ10, one very important issue that has received relatively little attention is the question of supplement quality and bioavailability. Because nutritional supplements are not regulated in the UK in the same way as prescription medicines, there is no legal quality requirement relating to defined levels of active substances and product stability; supplements may therefore, for example, contain lower levels of CoQ10 per capsule than stated on the product packaging. The best way to avoid this problem is therefore to use a CoQ10 supplement that has been manufactured to pharmaceutical standards.

There are currently more than 300 randomised controlled clinical trials relating to CoQ10 listed on Medline, including 70 on heart disease, 30 on diabetes, 10 on renal disease and six on liver disease. Whilst the majority of such studies have reported significant benefit of CoQ10 supplementation (particularly in heart disease), some studies have reported no significant benefit. Such disparity may result from a number of factors, including insufficient CoQ10 dosage or treatment duration, and inter-individual variation in the ability to absorb CoQ10, but particularly from inadequate bioavailability, especially where CoQ10 blood levels have not been determined pre- and post-supplementation. Because of the molecular characteristics of CoQ10, bioavailability is intrinsically low. As a lipid soluble substance, bioavailability is optimized via the use of a carrier oil such as soy oil; however, the key factor in determining bioavailability is the efficient dispersion of CoQ10 crystals formed during the yeast fermentation manufacturing process [68]. Similarly, there are currently some 40 meta-analyses relating to CoQ10 listed on Medline, including 15 on heart disease and three on diabetes. Some of these studies have reported a lack of evidence to support the use of CoQ10 supplementation in these various indications; for example, the Cochrane review by Madmani et al. [69] concluded that there was insufficient evidence to support the use of supplemental CoQ10 in heart failure, although this study did not include data from the Q-SYMBIO and KiSEL-10 clinical trials. However, the majority of meta-analyses have reported significant benefit of CoQ10 supplementation in the above indications; recent examples include those relating to cardiovascular disease [70,71,72] and diabetes [73].

Another factor affecting efficacy is the relative bioavailability of the ubiquinone and ubiquinol forms of CoQ10. Some supplement manufacturers have claimed that the ubiquinol form is more bioavailable, based on the concept that absorption of CoQ10 into enterocyte cells was thought to require reduction of ubiquinone to ubiquinol, and presentation of supplemental CoQ10 in ubiquinol form facilitated this absorption, particularly in patients with malabsorption disorders. However, work by Judy [74] demonstrated that reduction of ubiquinone to ubiquinol takes place within the lymphatic system rather than during absorption by enterocytes; in addition, during the time of transit from the stomach to the small intestine (typically 2–5 h), CoQ10 in reduced form would be oxidised to ubiquinone, a process taking approximately 80 minutes in simulated gastric conditions [74]. In the study by Lopez-Lluch et al. [68] in human subjects, the bioavailability of CoQ10 in ubiquinone form, when properly dispersed in carrier oil, was approximately twice that of the corresponding ubiquinol form; thus, it is the crystal dispersion status, rather than redox status, of the CoQ10 that essentially determines bioavailability.

## 7. Conclusion

In summary, we have reviewed evidence from randomised controlled clinical trials as to how oral supplementation with CoQ10, either alone or in tandem with selenium, can significantly reduce mortality risk from cardiovascular disease in normal elderly subjects, or in those with heart failure. In addition, supplementary CoQ10 may reduce mortality risk in patients with type II diabetes, chronic kidney disease or liver disease, both by beneficial effects on the primary disease process in these tissues, or on cardiovascular dysfunction secondary to these disorders.
